# Cognitive, Neuropsychological and Biological Effects of Oxygen–Ozone Therapy on Frailty: A Study Protocol for a 5-Week, Randomized, Placebo-Controlled Trial

**DOI:** 10.3390/jpm14080795

**Published:** 2024-07-27

**Authors:** Catia Scassellati, Cristian Bonvicini, Miriam Ciani, Roberta Zanardini, Evita Tomasoni, Valentina Saletti, Ilaria Passeggia, Monica Almici, Ilaria Pagnoni, Antonio Carlo Galoforo, Mario Costa, Mara D’Onofrio, Antonino Cattaneo, Cristina Geroldi

**Affiliations:** 1 Biological Psychiatry Unit, IRCCS Istituto Centro San Giovanni di Dio Fatebenefratelli, 25125 Brescia, Italy; c.scassellati@fatebenefratelli.eu; 2 Molecular Markers Laboratory, IRCCS Istituto Centro San Giovanni di Dio Fatebenefratelli, 25125 Brescia, Italy; robertazanardini@gmail.com; 3 Department of Biomedical Sciences, University of Modena and Reggio Emilia, 41121 Modena, Italy; miriam.ciani@unimore.it; 4 Laboratory Alzheimer’s Neuroimaging and Epidemiology, IRCCS Istituto Centro San Giovanni di Dio Fatebenefratelli, 25125 Brescia, Italy; etomasoni@fatebenefratelli.eu (E.T.); vsaletti@fatebenefratelli.eu (V.S.); ipasseggia@fatebenefratelli.eu (I.P.); 5 Clinical Trial Service, IRCCS Istituto Centro San Giovanni di Dio Fatebenefratelli, 25125 Brescia, Italy; malmici@fatebenefratelli.eu; 6 Neuropsychology Unit, IRCCS Istituto Centro San Giovanni di Dio Fatebenefratelli, 25125 Brescia, Italy; ipagnoni@fatebenefratelli.eu; 7 Oxygen-Ozone Therapy Scientific Society (SIOOT), 24020 Gorle, Italy; antonio.galoforo@gmail.com; 8 Institute of Neuroscience, Italian National Research Council (CNR), 56124 Pisa, Italy; costa@in.cnr.it; 9 Laboratory of Biology “Bio@SNS”, Scuola Normale Superiore, 56124 Pisa, Italy; antonino.cattaneo@sns.it; 10 European Brain Research Institute (EBRI) Rita Levi-Montalcini, 00161 Rome, Italy; mara.donofrio@ebri.it; 11 Alzheimer’s Unit, Memory Clinic, IRCCS Istituto Centro San Giovanni di Dio Fatebenefratelli, 25125 Brescia, Italy; cgeroldi@fatebenefratelli.eu

**Keywords:** cognitive frailty, frailty, randomized double-blind clinical trial, oxygen–ozone therapy, neuropsychological markers, biological markers

## Abstract

Cognitive frailty (CF) is a heterogeneous syndrome that is becoming one of the most serious health problems as the world’s population age is increasing. Elucidating its biological mechanisms as well as prevention and treatments is becoming increasingly significant, particularly in view of the associated health costs. We presented the study protocol of a research project funded by the Italian Ministry of Health (grant number RF-2016-02363298) aiming to investigate the cognitive and neuropsychological effects of a 5-week treatment with therapy based on the regenerative properties of ozone (O_3_) in a cohort of subjects stratified according to CF scores. We also studied the potential effects of O_3_ on blood-based biomarkers indicative of specific biological systems that may be altered in CF. Seventy-five older persons were recruited and randomly assigned to receive the active treatment (150 cc of oxygen-O_2_-O_3_ mixture at the concentration of 30 µg of O_3_ per cc of O_2_), O_2_, or the placebo (air) for 5 weeks. The main endpoints were the change in the scores of clinical scales from baseline (T0) to weeks 3 (T3), 9 (T9), and 15 (T15) after treatment and the change in biomarker levels resulting from transcriptomics, proteomics, and metabolomic patterns at the same times. The positive results from this study could have important clinical implications.

## 1. Introduction

Frailty is a multidimensional geriatric syndrome characterized by increased vulnerability to stressors as a result of the reduced functional capacity of different physiological systems [1,2,3,4]. Consequently, adults with frailty may be subject to frequent falls, may be disabled and immobile, may have a lower-quality life, and can require hospitalizations [5]. It has been estimated that the prevalence of frailty ranges from 4.0 to 59.1%, detected among community-dwelling older individuals aged 65 and older [6,7]. 

In addition to frailty in a general sense, there is another field that deserves attention in the context of aging, which is that of cognitive impairment. Frailty and cognitive impairment co-occur together and are complicatedly related [8], determining significant disability, reduced quality of life, and higher morbi-mortality [9]. In April 2013, the International Academy of Nutrition and Aging (IANA) and the International Association of Gerontology and Geriatrics (IAGG) agreed that “cognitive frailty” (CF) is a simultaneous state of both cognitive impairment (clinical dementia rating [CDR] = 0.5) and physical frailty (frailty phenotype) in older individuals without a definite diagnosis of dementia independent of other frailty dimensions (e.g., social, psychological and oral frailty) [10]. Although the definitions and assessments of CF have undergone some changes over time, older adults with both physical frailty and cognitive impairment are shown to be at higher risk of adverse health outcomes, such as death, disability, and hospitalization, than those with either condition alone [11,12,13]. Physical frailty can accelerate cognitive impairment as well as increase the risk of mild cognitive impairment (MCI) and dementia [14,15]. Particularly, a specific network named the salience network (SN) is functionally impaired in MCI or dementia patients [16,17]. The SN nodes (right anterior insula (RAI), left anterior insula (LAI), and combined left/right anterior cingulate cortex (ACC)) make up a key cortical network that detects and integrates responses to salient stimuli. AI regions of SN are functionally involved in coordinating activities in many cognitive processes, such as the reorientation of attention and switching among cognitive resources; ACC is primarily implicated in increased cognitive controls and conflict monitoring and regulates neural activities in association with AI regions during difficult tasks. In general, SN has been implicated in modulating the switch between the internally directed cognition of the default mode network (DMN) and the externally directed cognition of the central executive network (CEN).

Nine percent is the estimated prevalence of CF in adults aged 65 years and older, and it is common in older women living in the community [18].

The underlying molecular mechanisms of CF remain unclear. However, based on the close association seen between physical frailty and cognitive impairment, several review articles suggest that impaired hypothalamic–pituitary axis stress responses, energy homeostasis and mitochondrial dysfunctions, endocrine dysregulation, oxidative stress, nutritional and metabolomic factors alterations, gut dysbiosis, and vascular risk factors and diseases represent the potential underlying common mechanisms among these conditions [19]. Importantly, the phenotype of frailty has also been associated with underlying inflammatory mechanisms. A recent review [20] supported the potential role of some biomarkers such as, for instance, C-reactive protein (CRP) as an inflammatory marker and as a prognostic index in this syndrome.

For these multifaced underlying causes, accumulating evidence indicates the need for comprehensive geriatric assessment that helps to better characterize the CF etiopathogenesis, but also to develop, at the same time, new individualized mono-domain interventions to prevent adverse health outcomes for older adults with CF.

Since 1840, from its discovery, ozone (O_3_) and, consequently, oxygen (O_2_)-O_3_ therapy has expanded into the medical field, giving rise to compelling research in the recent decades which validates its clinical value [21]. Several papers [22,23,24] emphasized its relevant medical features, such as virucidal and bactericidal effects, inflammatory modulation, and circulatory stimulation, with considerable applications in several medical fields [25]. O_3_ is considered one of the most powerful oxidizing molecules in nature [26]. Indeed, its function is strictly associated with its power to directly interact with lipoproteins, phospholipids, viral capsids, and bacteria envelopes. Particular attention was recently paid to further specific O_3_ activities related to the cellular and humoral immune system which contribute to the proliferation of immunocompetent cells and the synthesis of immunoglobulins as well as biologically active substances (interleukins, leukotrienes, and prostaglandins). In this sense, O_3_ also seems to activate macrophage function and increase the sensitivity of microorganisms to phagocytosis. Finally, it has also been reported that O_3_ can interact with blood cells or other elements of the circulatory system, which would have beneficial properties to O_2_ metabolism, the antioxidant defense system, cellular energy, and microcirculation [26].

If, on the one hand, experimental evidence provides a solid preclinical rationale for the application of O_2_-O_3_ therapy to improve cognitive profiles in aging [27,28,29,30], clinical studies supporting its potential beneficial effects on cognition in humans are not available.

Here, we presented the study protocol of a research project funded by the Italian Ministry of Health (grant number RF-2016-02363298). The main aims of this research project are (1) to clarify the potential cognitive and neuropsychological effects of a 5-week treatment with O_2_-O_3_ therapy on older adults with frailty; (2) to elucidate the potential effects of this therapy on blood-based biomarkers associated with transcriptomics, proteomics, and metabolomics patterns. We describe the protocol study by dividing the Material and Methods section into two parts, where we specify what steps have been performed (PART 1) and what steps will need to be performed (PART 2). 

## 2. Materials and Methods

The study is a randomized, double-blind, placebo-controlled, 5-week trial to assess the cognitive, neuropsychological, and biological effects of O_2_-O_3_ therapy on 75 older adults stratified according to CF scores. The study is being performed at the IRCCS Centro San Giovanni di Dio Fatebenefratelli, Brescia, Italy. In Table 1, we show the activities and timelines of the study.

As this is an ongoing trial, we divided this section into two parts where we specify what steps have been performed (PART 1) and what steps will need to be performed (PART 2).

### 2.1. PART 1

#### 2.1.1. Trial Design and Setting

Participants have been recruited among those who have already had access to the Translational Memory Clinic of the IRCCS Centro San Giovanni di Dio Fatebenefratelli, but also by several methods, including advertisements in local newspapers, flyers located at our institute, and meetings in different cultural centers for the elderly. During the clinical trial for screening, we facilitated retention by regular contact and reminders in appointments and in the testing sessions.

#### 2.1.2. Ethics Approval

The protocol (version 3.0) was approved by the local ethical committee, the Ethics Committee of the IRCCS Centro San Giovanni di Dio Fatebenefratelli (rif. Parere 40-2018). At the time of face-to-face screening, all participants provided written informed consent to the study protocol to a member of the research team. The research was carried out in accordance with the International Conference on Harmonization of Good Clinical Practice (GCP/ICH) guidelines and was conducted in line with the principles of the Declaration of Helsinki.

#### 2.1.3. Inclusion and Exclusion Criteria

Inclusion and exclusion criteria have been applied to this trial. The inclusion criteria were that the subjects had to have an age range of 60–85; a diagnosis of amnesic or non-amnesic cognitive disorder, reported by the subjects themselves or by family members; an MMSE score ≥ 24/30; pharmacological therapy for the treatment of stable cognitive or behavioral disorders for at least 3 months at the time of enrolment; no presence of dementia or ascertained Alzheimer’s disease detected by the most advanced diagnostic techniques; function intact, or functional reduction not related to the cognitive problem. We excluded subjects who were affected by disabling vision or hearing impairments; affected by known psychosis or psychiatric illness, alcohol or drug abuse; affected by uncontrolled hyperthyroidism or by Glucose-6-Phostate Dehydrogenase Deficiency (G6PD)-Favism; and those who considered the methodology for rectal insufflation invasive. 

#### 2.1.4. Study Schedule

**Figure 1:**
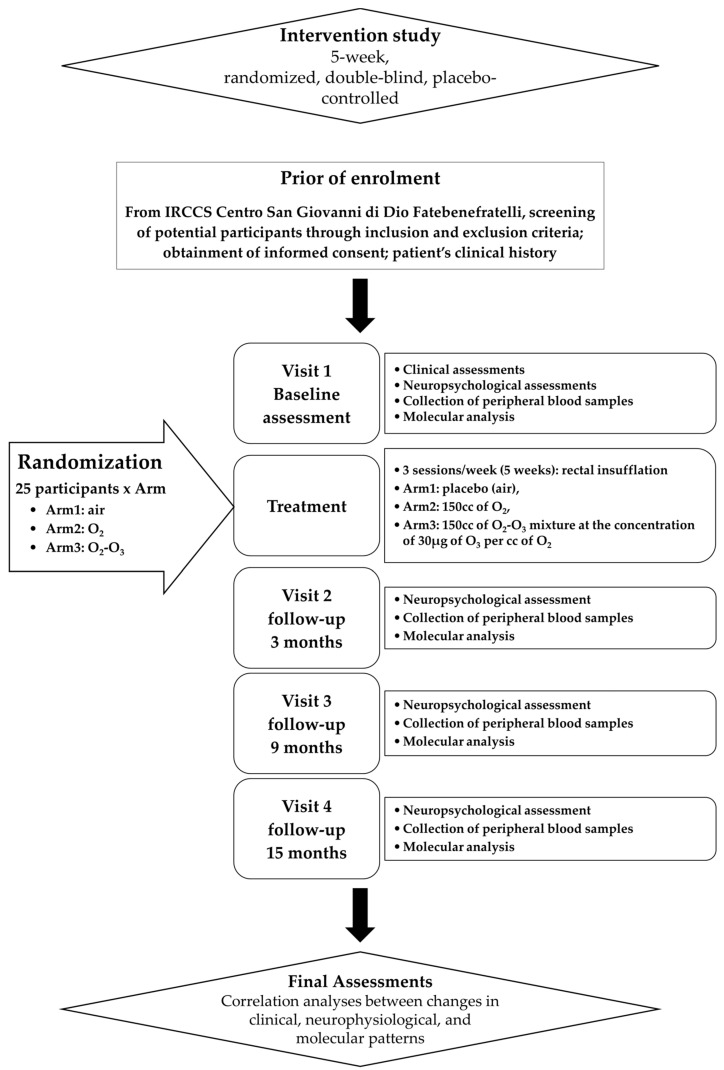
Flowchart of clinical trial.

In Figure 1, the study schedule of the following trial protocol is shown.

Participants were invited to undergo a face-to-face clinical assessment and neurocognitive batteries. In addition, subjects were asked about their socio-demographic status, their lifestyle, and their diet habits. Body Mass Index (BMI) was calculated too. The Everyday Memory Questionnaire (EMQ) was administrated to evaluate the presence of Subjective Memory Complaints (SMCs), whereas the Cognitive Reserve Index questionnaire (CRI-q) was used to measure cognitive reserve. Symptoms of depression were evaluated with the Geriatric Depression Scale (GDS), whereas physical health in general was estimated by the number of chronic diseases, by the Cumulative Illness Rating Scale (CIRS index), and by the number of drugs. Moreover, functioning skills in daily life were valued through the Barthel Index for Activities of Daily Living (Barthel index), Tinetti, and Instrumental Activities of Daily living (IADL) scales, in order to investigate the functional impact of any cognitive difficulties. Finally, an assessment of physical activity in daily life was performed. 

Specific scales/tools were used to assess the frailty status: the “Italian Frailty Index” (IFI) [31], an Italian version of the frailty index based on the accumulation of age-related deficits; the Frailty Instrument (FIt) for primary care based on the Survey of Health, Aging and Retirement in Europe (SHARE-FIt) [32], valuating five items (i.e., grip strength and four self-reported items: fatigue, loss of appetite and/or eating less than usual, difficulties climbing stairs and/or walking 100 m, and low level of physical activity); and the CDR scale to quantify the severity of symptoms of dementia.

A baseline neuropsychological visit, scheduled within a mean of 20 days of the face-to-face clinical assessment, was administered to all eligible participants, who were then allocated to one of the three intervention groups. A blood sample was collected for biomarker measurement from all subjects. Follow-up neuropsychological visits included (i) a visit 3 months after treatment (T3); (ii) a midpoint visit 9 months after treatment (T9); and (iii) an endpoint visit 15 months after treatment (T15). In each follow up, we collected blood samples for biomarker measurement.

#### 2.1.5. Adaptive Randomization

Subjects were divided into three groups with different levels of cognitive frailty, defined according to tertiles of distribution of the scores of tests for the evaluation of memory skills (Story recall, Rey Auditory Verbal Learning Test—RAVLT immediate and delayed recall, and Rey–Osterrieth Complex Figure—ROCF Recall), attention skills (Trail Making Test part A and part B, TMT-A, TMT-B, Rey–Osterrieth Complex Figure—ROCF copy), and MMSE and GDS scores.

Participants were randomly assigned to the active treatment group (O_2_; O_2_-O_3_) or the placebo group (air), balanced for CF, so that each treatment arm accommodated a similar proportion of subjects in each tertile. The definition of the tertiles of CF was performed at different times, over a period of 12 months, on groups of 12/16 subjects, as enrollment progressed and before starting treatment. Being a double-blind study, both the researchers of the research team and the participants remained blinded to the treatment assignment until the completion of the study, while the medical doctor performing the treatment knew the treatment group, but not tertile of fragility. During recruitment, the medical doctor performing the treatment periodically re-evaluated the size of the treatment subgroups based on the recruitment and dropout rate.

#### 2.1.6. Compliance and Adverse Effects

Compliance was assessed by instructing the participants to note/record any medication (new or not) intake and/or adverse events that might have happened during the course of the trial. We closely monitored the potential adverse events and, for this, we instructed the participants to immediately contact the research team in the case of unexpected medical care visits or serious adverse events/hospitalization. Reasons for discontinuation have been recorded.

#### 2.1.7. Primary and Secondary Endpoints

The primary endpoint was the change in the scores of the clinical IFI or SHARE-Fit scales from baseline to the T3, T9, and T15 trial period after treatment.

The secondary endpoint was the change in the molecular levels (RNA and proteins) from baseline over the T3, T9, and T15 trial period after treatment.

#### 2.1.8. Intervention

***Active.*** The medical specialist inserted a small CH14-18 catheter into the rectum and gently administered a total amount of 150 cc of O_2_-O_3_ mixture at the concentration of 30 µg of O_3_ per cc of O_2_ over a 5–10 min period while the patient was laying on his/her left side. The patient was asked to refrain from passing gas or having a bowel movement for at least 30 m. This procedure is known as rectal insufflation. It was performed for 3 sessions/week (5 weeks). By the same approach, subjects were insufflated with 150 cc of pure O_2_.

***Placebo.*** By the same methodology and times and sessions, 150 cc of air was insufflated as a placebo treatment. 

#### 2.1.9. Data Collection

***Neuropsychological batteries*.** All participants were tested at each visit with the Mini Mental State Examination (MMSE) [33] to assess the global cognition, and several cognitive tests were selected to assess a broad range of cognitive abilities: RAVLT, immediate and delayed recall, ROCF Recall, TMT-A, TMT-B, ROCF copy, action and object naming subtests from the Battery for the Assessment of Aphasic Disorders (B.A.D.A), Raven’s Colored Progressive Matrices (CPM47), the Free and Clued Selective Reminding Test (FCSRT), and the Face–Name Association task (FNAT) [34]. In addition, baseline assessment (time = T0) included the administration of a digit span forward and digit span backward test. Story recall, phonemic verbal fluency (FPL) and semantic verbal fluency (FPC) tasks, the Token Test Auditory sentence comprehension subtest from BADA, and the De Renzi ideomotor apraxia test (right and left upper limb) were also used [35]. 

***Biological measures*.** Fasting venous blood samples were collected in the morning at the baseline (T0) and at follow-ups T3, T9, and T15 for the evaluation of alterations/modulations at the molecular level before and after treatment. 

**Proteomic analyses.** We used anticoagulant-free tubes and EDTA tubes to collect serum and plasma samples, respectively. Serum tubes were kept at room temperature for 1 h followed by 1 h at 4 °C before serum separation by centrifugation (2000× *g* for 10 min). Plasma tubes were immediately centrifuged at 2000× *g* for 10 min. Both serum and plasma samples were stored at −80 °C until the time of the assay. 

An assay was also performed for C-reactive protein (CRP) on serum samples from each subject at time T0.

**Mass spectrometry.** To extract metabolites, frozen serum samples were thawed at 4 °C, and 145 µL samples were transferred into 1.5 mL low protein binding tubes. Then, 5 µL of ESI Tuning Mix and 300 µL of extraction solvent (ACN, acetonitrile) were added to 145 µL of serum sample, mixed by vortex for 30 s, and centrifuged at room temperature and 13,000 rpm (15,871× *g*) for 10 min. Then, ~400 µL supernatant samples were transferred into 1.5 mL low protein binding tubes, and the extracts were dried in a vacuum concentrator at 30 °C. Sample-dried pellets were stored at 4 °C overnight (16 h) and reconstituted the next day prior to analysis. For reconstitution, 50 µL of ACN/H2O (20:80, *v*/*v*) was added to the pellet, mixed by vortex for 60 s, and then transferred to vials for LC-MS analysis. A procedure blank was generated identically without serum and used later to remove the background ions. Equal volumes of serum from all biological test samples were pooled as the quality control (QC) sample for the UHPLC–QqTOF–MS analysis. The QC samples were used to assess the reproducibility and reliability of the LC–MS system and to determine the repeatability of a metabolite feature.

**Transcriptomic analyses.** Peripheral blood samples were collected in PAXgene Blood RNA Tubes (PreAnalytiX, Qiagen, Valencia, CA, USA) and stored at −80 °C before RNA extraction. Total RNA was isolated according to the manufacturer’s instructions. The NanoDrop 2000 Spectrophotometer (Thermo Fisher Scientific, Waltham, MA, USA) was used for assessing RNA purity, and 260/230 ratios were evaluated. The Agilent 2100 Bioanalyzer by Eukaryote total RNA 6000 nano Kit (Agilent Technologies, Santa Clara, CA, USA) was used to determine concentrations and integrity. Samples with an integrity value lower than 7.0 were deleted.

#### 2.1.10. Data Management

The study investigators had accessibility to data collection forms and protocols in a secured shared drive. Moreover, they entered the data collected at the visits electronically, and then, the PI checked these data. The participant data were protected by password, so that all information remained confidential, and only study investigators had access to these data.

In addition, participants have been identified by a coded identification number that, to maintain participant confidentiality, has been used to identify all laboratory specimens, data collection, and administrative forms. The study coordinators and PI had access to the final trial dataset. The PI oversaw intrastudy data sharing.

#### 2.1.11. Data Monitoring

All aspects of data monitoring were overseen by the study coordinators with the assistance of the PI. Moreover, we expected subjects to be exposed to minimal risks during participation in this trial; thus, no interim analyses have been performed.

#### 2.1.12. Statistical Analysis

***Sample size*.** The power analysis was performed by the analysis of the univariate linear regression test. Using the accuracy of the model IFI scale as a multiple partial coefficient (R^2^) and the neuropsychological tests as fixed predictors, we found a power effect of 0.92 related to our population of 76 subjects. Similar findings (power effect of 0.80) were found when we considered the SHARE-Fit scale.

### 2.2. PART 2

#### 2.2.1. Molecular Analyses

Blood sample material will be used to perform transcriptomic, proteomic, and metabolomic studies.

***Proteomic analyses*.** We plan to perform a cytokines and growth factors panel to be analyzed through automated the ELISA Platform (Bioplex, Biorad, Hercules, CA, USA).

***Mass spectrometry***.

**UHPLC–QqTOF–MS analysis.** Serum metabolic profiling analysis will be conducted using a UHPLC–MS system with a Bruker compact QqTOF MS (Bruker Daltonics, Billerica, MA, USA) and a Dionex UltiMate 3000 Rapid Separation LC (Thermo Fisher Scientific, Waltham, MA, USA) equipped with an Acquity UPLC BEH C18 (300 Å, 2.1 × 150 mm, 1.7 μm) column (Waters). MS parameters will be set up using microToF 3.4, ESI Compass 1.3, and HyStarPP 3.2 SR4 software (Bruker Daltonics, Billerica, MA, USA). A 10 µL aliquot of samples will be injected into the HPLC column, and elution will be performed at a flow rate of 0.4 mL/min using a gradient elution program as follows: 0 min, 5% B; 2 min, 5% B; 7 min, 40% B; 9 min, 80% B; 9.1 min, 100% B; 14 min, 100% B; 14.1 min, 5% B; 20 min, 5% B. The column temperature will be set up at 60 °C. The total running time will be 20.1 min. Mobile phase A will consist of 0.1% CH2O2 (formic acid) in H_2_O, while mobile phase B will consist of 96% EtOH. Ionization will be performed using an electrospray ionization (ESI) source, operating in positive mode. ESI source conditions will be set up as follows: capillary voltage, 4.0 kV; dry gas (N2) flow, 8.0 L min^−1^; dry gas (N2) temperature, 300 °C; nebulizer pressure, 3.0 bar. The mass scan range will be set from 200 to 950 *m*/*z*. The instrument will be calibrated prior to the analyses using 10 mM sodium formate.

**Data processing and analysis.** UHPLC–MS raw data files (.d) will be converted to mzML format using ProteoWizard (version 3.0.23339). The open-source MZmine 3 software (version 3.6.0) will be used to generate a peak table containing *m*/*z*, retention time (RT), and intensity for the peaks (also called features). Parameters for feature detection will be set up as follows: scan to scan accuracy, 0.005 *m*/*z*; minimum intensity, 1.0 × 10^3^; minimum peak width, 5 scans; minimum RT search range, 0.05 min; noise level, 1.0 × 10^2^. The retention time scan window will be limited to 5–15 min. In addition, 13C-related isotope peaks will be removed if peaks are ≤0.01 min apart, their *m*/*z* difference is ≤0.001 or 3 ppm, or if a monotonically decreasing trend in the isotope pattern is detected, retaining the most intense peak. The alignment of peaks and gap-filling will be performed by setting the following parameters: sample-to-sample *m*/*z* tolerance, 0.05 *m*/*z* or 50 ppm; RT tolerance, 0.4 min. Redundant peak entries due to misaligned feature list rows will be removed if two peaks are ≤0.05 min apart and their *m*/*z* difference is ≤0.05 or 10 ppm. Background peaks will be removed if the intensity in the biological samples is <3-fold of that in the procedure’s blank samples. Features not detected in at least 75% of QC samples will be excluded from the final table. The resulting peak table will be exported to a .csv file. Normalization of the dataset will be performed by applying the total intensity in an analysis (or sum of bucket values in analysis) normalization method. From the normalized data matrix, features with RSDs higher than 30% in the QC samples will be filtered out before proceeding with statistical analyses. We plan to perform a metabolic profile for each subject at baseline and after treatment.

***Transcriptomic analyses*.** We plan to perform gene expression profiling using the standard protocol for Agilent one-color gene expression microarray [36,37].

#### 2.2.2. Statistical Analysis

***Data analysis*.** We will present as means and standard deviations the descriptive statistics on cognitive and biological markers in the three treatment groups. The association between categorical variables will be analyzed using the Chi-square test.

The Automatic Linear Modeling (ALM) model that is based on a machine learning approach to find the best predictive model using available data will be used. A set of neuropsychological/biological values will be analyzed as independent input variables, while the scores of IFI or SHARE-FIt scales will be analyzed as continuous output (target) (dependent) variables. Longitudinal comparisons will be performed using univariate analysis, where the clinical parameters relating to the IFI and SHARE-FIt scales will be entered as dependent variables, the significant values obtained from ALM as covariates, and time and treatment as fixed factors. For the metabolomic analysis, PCA analysis (Principal Component Analysis) and a univariate analysis will be performed to evaluate the effects of the treatment on the presence/distribution of the metabolites. 

With the aim to correlate any clinical/cognitive improvements with molecular profiles to identify markers associated with response to treatment, the generalized linear mixed model (GLMM) will be used where the biological values and those relating to neuropsychological tests and the times of follow-up will be analyzed as independent input variables, while the values relating to the IFI or SHARE-FIt scales will be analyzed as weight variables and the treatments as (dependent) variables. “Air treatment” will be used as a comparison variable. All these analyses will be performed using SPSS ver. 29.

Differentially expressed genes will be identified using R-Bioconductor by applying both a fold change and p-value threshold (R-Limma test *p* value < 0.05; |Log2 fold-change ratio| > 1.0). By Fisher’s exact test, we will analyze the categories and functional pathways of upregulated or downregulated genes, using all genes in the array as the “reference” genome, or through the use of Ingenuity Pathway Analysis (IPA, Qiagen, Venlo, The Netherlands) to infer alterations in biological pathways and functions. 

#### 2.2.3. Dissemination

By using peer-reviewed journals and scientific conference presentations, we will publish the results obtained from this trial to be considered by scientific investigators and healthcare professionals. The authorship requirements will adhere to the guidelines provided by the scientific journals. In addition, individual trial outcomes will be sent to participants with their consent. We will transmit the findings obtained from this study to the general population by using different and specific tools.

## 3. Discussion

This article presents the rationale and the materials/methodologies of this ongoing pilot clinical trial that has the aim of analyzing the cognitive and biological effects of O_2_-O_3_ therapy on the clinical/neuropsychological and molecular patterns in older adults with CF. This study protocol is a research project funded by the Italian Ministry of Health, grant number RF-2016-02363298. Molecular analyses and their correlations with clinical and neuropsychological profiles and with different treatments with O_2_-O_3_ therapy are currently ongoing.

Currently, specific treatments are not completely available for elderly individuals affected by CF, subjects who are potentially at risk of developing cognitive impairment and dementia. Consequently, the implementation of further potential strategic interventions may represent a useful approach to promote cognitive health and ultimately prevent cognitive decline.

O_3_ is a triatomic gaseous molecule used as a powerful oxidant in medicine for more than 150 years [38]. It has been demonstrated that O_3_ concentration and effects do not follow a linear relationship: very low concentrations could have no effect, whereas very high concentrations can lead to contrary effects to those produced by lower/middle concentrations [39]. At least 65 findings reviewed by Scassellati et al. [40] demonstrated the preconditioning/postconditioning effects of O_3_ on endogenous pro-antioxidant mechanisms in vivo on animal models and in vitro on cells. In this direction, we will test for the first time whether there is an effect of this therapy on frailty in a sample of aging subjects screened for CF scores. The rationale on which we based our hypothesis comes from some preclinical studies that demonstrated how O_3_ could be beneficent during aging through the regulation of oxidant–antioxidant homeostasis. In particular, Safwat et al. [27], performing experiments in aged rats, demonstrated that O_3_ reduced liver and kidney damage, increasing the reduced hepatic and renal glutathione (GSH) contents, as well as normalizing hepatic glutathione peroxidase (GSH-Px) enzyme activity. In another study [30], the administration of O_3_ in aged rats normalized the reduced GSH content, adenosine triphosphate/adenosine diphosphate ratio, mitochondrial superoxide dismutase (SOD), and complex IV (cytochrome-c oxidase) activities. Moreover, O_3_ improved the glutathione redox index (GSHRI), complex I (NADH-ubiquinone oxidoreductase), and mitochondrial nitric oxide synthase (mtNOS) activities and attenuated the rise in malondialdehyde (MDA) and mitochondrial phosphatidylcholine (PC) levels. In the same animal model of aged rats, another work [29] demonstrated that O_3_ decreased the lipid and protein oxidation markers as well as the lipofuscin deposition and restored both GSH levels in brain and heart tissues and GSH-Px activity in the heart tissue. O_3_ also mitigated age-associated energy failure in the heart and the hippocampus, improved cardiac cytosolic Ca(2+) homeostasis, and restored the attenuated Na(+), K(+)-ATPase activity in the hippocampus of these rats. A further study [28] demonstrated that O_3_ administration ameliorated the behavioral and pathological deterioration of APP/PS1 transgenic mice and reduced the level of amyloid-β precursor protein (APP).

By using a certificated O_3_ generator device, a gas mixture of O_2_-O_3_ obtained from the modification of medical-grade O_2_ is employed in the medical setting [41]. The therapeutic range of O_3_ has been precisely calculated, starting from the basic mechanisms of action of O_3_ in blood, and it has been found to be 10–80 μg/mL of O_3_ in blood [42]. Different and main routes of application with relative concentrations of O_3_ have been widely described by Schwart-Tapia et al. [42]. The side effects are minimal: the World Federation of Ozone therapy (WFOT) estimates the incidence of complications at 0.0007%. Moreover, given that most patients report a feeling of wellness and euphoria during the cycle of the treatment, O_3_ treatment is perfectly tolerated, and this explains why the compliance of patients remains excellent throughout the years.

To assess the frailty/CF, we used IFI as well as SHARE-FIt scales, along with CDR scores.

As reported in Sugimoto [19], there are some important issues that lead to difficulty in adapting the CF concept to clinical practice. Indeed, many studies use varied CF models and assessment tools with varied definitions of CF, making the comparison of findings across studies challenging. Therefore, a valid operational definition and an assessment tool that detect older adults at risk for reversible conditions could need to be established. Another challenge is represented by the lack of an appropriate tool for the evaluation of cognitive impairment. In this direction, we have enriched our clinical valuations by implementing a complete battery of neuropsychological tests which have been used in our trial.

The secondary endpoint of the trial is the change in biological markers levels in terms of proteomics, transcriptomics, and metabolomics. As the syndrome is multidimensional, the identification of biomarkers in CF is a complex process. In the context of the neuroimaging field, several recent papers agreed that CF is associated with loss of structure in specific brain regions such as the thalamus and hippocampus, as well as changes in white matter hyperintensity [43]. At the metabolic level, a longitudinal study performed in a population of 7769 individuals included in the Doetinchem Cohort Study [44] evidenced that markers such as high-density lipoproteins, triglycerides, alanine aminotransferase, gamma-glutamyltransferase, albumin, uric acid, cystatin C, and creatinine were not predictive of CF. Contrarily, Royal and Plamen [45] observed that the levels of insulin-like growth factor 1 and IGF-binding protein 2 levels were found to be altered in patients with cognitive decline and physical frailty. Moreover, Sargent et al. [46] reported lower levels of vitamin E alpha tocopherol, omega-6 and 3, and albumin in subjects with CF. In addition, the same authors found a further significant association with lower levels of low- and high-density lipoproteins. Finally, the focus on microbiota is a new further aspect in the development of new age-related biomarkers. For instance, He et al. [47] demonstrated that CF patients showed elevated levels of trimethylamine N-oxide, a stable metabolite of the intestinal microbiota.

In this study, we will apply a high-throughput -omic approach that will allow us to analyze the whole transcriptome and proteome in order to identify potential known as well as new biomarkers associated with the response to O_2_-O_3_ therapy treatment and, consequently, new therapeutic targets for O_3_. We thus expect that O_3_ can potentially influence biological systems linked to the immune–inflammatory system, the stress response, and oxidative stress, regeneration processes which are altered in the CF. In particular, those genes that will be differentially expressed before and after treatment and that thus will be modulated by the therapy will be verified at the biochemical level along with a panel of proteins (proteomics) that will include cytokines (i.e., IL-1β, IL-6, TNFα), immune factors (i.e., MCP-1, RANTES, MIP1), and growth factors (i.e., VEGF, BDNF, NGFβ).

These -omic approaches along with bioinformatics will allow the identification of new molecular systems involved in the etiopathogenetic mechanisms of CF and potentially new mechanisms of action for the O_3_.

This ongoing trial has some strengths. The first is the length of the study: considering the three follow-up visits in the 15 months after treatment, it was long enough to expect to have more evidence on the trajectory of cognitive changes. Second, in this study, we will include an -omics biological corollary that will permit us to potentially identify new biomarkers associated with the response to treatment with O_2_-O_3_ therapy. Moreover, this will allow us to deepen possible new mechanisms of action of the O_3_ underlying the proposed treatment-associated benefits on cognition. On the other hand, although our sample has a power effect of 0.92/0.80, the results we will obtain should be confirmed in a larger population.

## 4. Conclusions

Positive results deriving from this project will allow future clinical studies starting from a larger available clinical cohort to be designed, with the main aim of testing O_2_-O_3_ therapy as a potential therapeutic strategy for the prevention of cognitive deterioration. Moreover, these data will allow public health organizations to plan non-pharmacological interventions for at-risk older individuals.

## Figures and Tables

**Table 1:** Activities and timelines of the RF-2016-02363298 study.

				Follow-Up		
Procedures	Screening	Baseline		T3 3 Months	T9 9 Months	T15 15 Months
**Weeks**	**−2 to T0**		**5**			
Informed consent	✓					
Medical history/documents	✓					
Inclusion/exclusion criteria	✓			✓	✓	
Randomization		✓				
Treatment (3 times × week)			✓			
Clinical scales		✓				
Neuropsychological scales		✓		✓	✓	✓
Blood sample collection		✓		✓	✓	✓
Molecular analyses		✓		✓	✓	✓
Bioinformatics				✓	✓	✓
AE/SAE (adverse events/serious adverse events)	✓		✓	✓	✓	✓
Complete Case Report Forms (CRFs)		✓		✓	✓	✓

## Data Availability

The datasets of raw data generated for this study are found in the Zenodo Data Repository (10.5281/zenodo.10805255) [48].
